# Plant–Microbe Interaction

**DOI:** 10.3390/biology10010015

**Published:** 2020-12-30

**Authors:** Aria Dolatabadian

**Affiliations:** Department of Plant Science, University of Manitoba, Winnipeg, MB R3T2N2, Canada; aria.dolatabadian@gmail.com

Plant–microbe interaction is a complex, dynamic and continuous process that is as old as plant colonization on Earth. Millions of years’ association of plants with microbes has formed an assemblage of host and non-host species, forming a discrete ecological unit called “holobiont”. In both natural and agricultural ecosystems, plants are regularly invaded by beneficial and pathogenic micro-organisms, mainly bacteria and fungi [1]. The beneficial interactions can be defined as some direct or indirect mechanisms such as nutrient transfer, performed by mycorrhizal fungi and rhizobia that associate with roots and provide plants with mineral nutrients and fixed nitrogen, respectively, direct stimulation of growth through phytohormones, antagonism towards pathogenic micro-organisms, and mitigation of stresses. On the other hand, the harmful interactions are detrimental to plants as the invading microbes may be saprophytic and cause necrotrophy in the colonizing plants. Therefore, deciphering plant–microbe interaction is a critical component in recognizing the positive and negative impacts of microbes on plants.

To date, numerous studies have been conducted to reveal the plant–microbe interaction processes, for example, how plants respond to microbial colonization and how microbial pathogens and symbionts reprogram plant cellular processes [2]. Nevertheless, many controversial issues exist about how plants differentiate between beneficial and pathogenic microbes or between different pathogen species or how gene regulatory networks and signal transduction pathways control these processes. Recently, it has been discovered that ligand-recognizing motifs in *Lotus japonicus* LysM receptors are significant determinants of specificity [3] so that *L. japonicus* plants use these small, well-defined motifs in receptor proteins to initiate differential signalling of immunity or root nodule symbiosis. Research findings also disclose that microbes found on/in plants tissue produce several different signals, including volatile organic compounds, hormones and hormone mimics, carbohydrate- and protein-based signals [4]. Microbes have carbohydrate- and protein-based signals classified as Microbe- or Pathogen-Associated Molecular Patterns (MAMPs or PAMPs) essential for microbial survival [5]. Based on their conservation and the fact that they are not synthesized in plant cells, plants have evolved different plasma membrane-localized Pattern Recognition Receptors (PRRs) that bind MAMPs and PAMPs and control plant immune responses. In response to PAMPs, plants trigger a defence response called PAMP-Triggered Immunity (PTI) or basal resistance, the first level of defence that restricts pathogen infection in most plant species [6]. During the invasion, microbes secrete effectors molecules which play roles as crucial elements of pathogenesis [7]. In response, plants have evolved resistance (R) genes encoding R proteins, making them recognize, directly or indirectly, some of these effectors (avirulence proteins). Recognition of a pathogen avirulence protein triggers a set of immune responses grouped under the term Effector-Triggered Immunity (ETI).

In legumes, the symbiotic association starts with mutual recognition of signal molecules, rhizobia perceive plant-derived flavonoids and produce a lipo-chitooligosaccharide signal (Nod factor). In return, legume plants perceive Nod factor, resulting in the activation of subsequent symbiotic reactions that lead to rhizobial infection and nodule organogenesis [8]. As regards arbuscular mycorrhizal association, recognition initiates by exchanging chemical signals between plant and fungi. Plants release strigolactone that stimulates spore germination and promotes hyphae growth where mycorrhizal factors, including lipo-chitooligosaccharides and chitooligosaccharides, are produced and recognized by plants to activate the signalling pathway of the symbiosis in the root [9]. 

Today, with the advent of high-throughput sequencing of plants and microbes’ genomes, novel technologies have offered new opportunities to unravel the mechanisms underlying microbes’ recognition, signal transduction and symbiosis establishment as well as a defence response in plants. For instance, identification of plants’ Resistance Gene Analogs (RGAs), including Nucleotide Binding Site-Leucine Rich Repeats (NBS-LRRs), Receptor-Like Kinases (RLKs) and Receptor-Like Proteins (RLPs), holds great promise for the development of resistant cultivars [10]. More than 34,000 RGAs were identified across Brassicaceae wild and domesticated species [11]. The study established a resource for the identification and characterization of RGAs in the Brassicaceae family. Kamal et al. [12] evaluated symbiosis gene copy number and distribution from a chromosome-scale *L. japonicus* Gifu genome sequence and showed that the symbiotic islands recently described in *Medicago truncatula* do not appear to be conserved in *L. japonicus*. Their research is a valuable resource for legume functional and comparative genomics. 

Understanding the molecular mechanisms of plant–microbe interaction would help develop innovative genetic engineering strategies of symbiosis, mutualism, and disease resistance through gene editing, RNA silencing, and other approaches. 

This Special Issue on plant–microbe interaction addresses both friendly and hostile plant–microbe encounters. It brings new experiential results and some advances to unravel the complexity of plant–microbe interactions and their role in plant community structure and function.

The Special Issue focuses on cutting-edge knowledge on beneficial and pathogenic plant and micro-organism interactions, variations in plant or microbes’ factors and plant defence mechanisms as well as plant immune system evolution and function, obtained from the application of physiological principles, genetics, and genomics, as well as bioinformatics and computational modelling approaches.

## Data Availability

Not applicable.
